# A Designed Experiments Approach to Optimization of Automated Data Acquisition during Characterization of Bacteria with MALDI-TOF Mass Spectrometry

**DOI:** 10.1371/journal.pone.0092720

**Published:** 2014-03-24

**Authors:** Lin Zhang, Connie M. Borror, Todd R. Sandrin

**Affiliations:** School of Mathematical and Natural Sciences, Arizona State University, Phoenix, Arizona, United States of America; Charité, Campus Benjamin Franklin, Germany

## Abstract

MALDI-TOF MS has been shown capable of rapidly and accurately characterizing bacteria. Highly reproducible spectra are required to ensure reliable characterization. Prior work has shown that spectra acquired manually can have higher reproducibility than those acquired automatically. For this reason, the objective of this study was to optimize automated data acquisition to yield spectra with reproducibility comparable to those acquired manually. Fractional factorial design was used to design experiments for robust optimization of settings, in which values of five parameters (peak selection mass range, signal to noise ratio (S:N), base peak intensity, minimum resolution and number of shots summed) commonly used to facilitate automated data acquisition were varied. *Pseudomonas aeruginosa* was used as a model bacterium in the designed experiments, and spectra were acquired using an intact cell sample preparation method. Optimum automated data acquisition settings (i.e., those settings yielding the highest reproducibility of replicate mass spectra) were obtained based on statistical analysis of spectra of *P. aeruginosa*. Finally, spectrum quality and reproducibility obtained from non-optimized and optimized automated data acquisition settings were compared for *P. aeruginosa*, as well as for two other bacteria, *Klebsiella pneumoniae* and *Serratia marcescens*. Results indicated that reproducibility increased from 90% to 97% (p-value
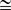
0.002) for *P. aeruginosa* when more shots were summed and, interestingly, decreased from 95% to 92% (p-value 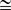
_ 0.013_) with increased threshold minimum resolution. With regard to spectrum quality, highly reproducible spectra were more likely to have high spectrum quality as measured by several quality metrics, except for base peak resolution. Interaction plots suggest that, in cases of low threshold minimum resolution, high reproducibility can be achieved with fewer shots. Optimization yielded more reproducible spectra than non-optimized settings for all three bacteria.

## Introduction

Matrix-assisted laser desorption/ionization time-of-flight (MALDI-TOF) mass spectrometry (MS) has emerged as a rapid and accurate technology to characterize bacteria at the genus and species levels [1]–[7]. Such characterization is based on unique mass spectra associated with different bacteria and obtained by analysis of whole cells or cellular extracts [2], [8]. Peaks represent biological molecules, typically proteins, that originate from cell surfaces, intracellular membranes, and ribosomes [9], [10], and thus are unique and constitute fingerprints. Mass spectra can be acquired either manually [11], [12] or automatically [12], [13], but automated data acquisition can enhance the high-throughput nature of this approach. Due to the rapidity and efficacy of this technique, there has been keen interest in application of MALDI-based approaches to characterize bacteria at the strain and subspecies levels [10], [14]–[18]. Strain level characterization is challenging because strains within a single species are often extremely similar and yield mass spectra with only subtle differences [18], [19]. Several studies have shown that spectra with poor quality and/or low reproducibility may confound bacterial identification and lead to misclassifications [8], [18]–[20]. Consequently, strain level identification is effective only when the reproducibility (i.e., similarity) of replicate spectra of individual strains exceeds that of spectra of unique strains of interest [21].

Because high quality and highly reproducible mass spectra are required to ensure reliable strain level characterization [22], [23], many efforts have been made to optimize data collection conditions to improve spectrum quality and reproducibility. Optimization strategies are generally divided into two categories: optimization of pre-analytical procedures and optimization of post-processing procedures. Many pre-analytical parameters have been reported to influence spectrum quality and reproducibility, including culture age [24], growth medium [25], matrix [26], solvent composition [27], and sample preparation and deposition method [18], [28]. Several of these parameters have been optimized using common univariate approaches in which one variable is changed at a time, and a series of experiments is conducted to determine the optimal condition for each parameter [17], [29]–[31]. Though often effective, optimization based on univariate approaches presents a number of limitations. First, optimization may be not universal, because few studies have tested the resulting optimal condition beyond the species or strains that undergo optimization. Second, univariate-based optimization procedures are time-consuming and labor intensive. Another drawback of univariate approaches is that it is difficult to estimate interaction effects of parameters on reproducibility. With regard to optimization of post-processing criteria (e.g. to optimize Bruker MALDI Biotyper score cutoffs to improve the percentage of bacteria correctly identified [32]), spectrum quality and reproducibility are generally not quantified.

We previously reported that automated data acquisition yielded less reproducible spectra than manual data acquisition [11]. To automate MALDI data acquisition, users specify threshold values of several parameters (e.g., base peak intensity, minimum resolution, signal to noise ratio (S:N), etc.) necessary for the automation algorithm to acquire spectra. We hypothesized that the lower reproducibility associated with automated data acquisition may be due to non-optimized values of data acquisition parameters [11]. In fact, it has been noted previously that automated acquisition settings of the MALDI-TOF mass spectrometer needed to be optimized for better performance of fingerprint-based approaches [2]. Further, effects of data acquisition parameters on reproducibility and quality have not been thoroughly investigated. For each of these reasons, we sought to optimize automated data acquisition. Preliminary work in our lab using univariate approaches did not markedly enhance spectrum reproducibility [11]. As a result, we chose a multivariable method, factorial design of experiments, to characterize and optimize automated data acquisition. Several studies have shown that this approach to statistical design of experiments is an efficient way to provide richer information and extract the maximum amount of information from the most economic number of experiments [33]–[36].

The specific objective of this study was to optimize automated data acquisition to yield spectra with reproducibility comparable to those obtained manually. *Pseudomonas aeruginosa*, which was shown previously to yield spectra of significantly lower quality and reproducibility when data were acquired via automation than when acquired manually [11], was used as a model bacterium in the designed experiments. Finally, the optimal combination of automated data acquisition parameters we obtained using *P. aeruginosa* was also tested with *Klebsiella pneumoniae* and *Serratia marcescens*. Both of these bacteria are Gram-negative bacteria that showed much lower reproducibility when data were acquired via automation than when acquired manually [11].

## Materials and Methods

### Bacteria and reagents

*Pseudomonas aeruginosa* (ATCC 27853), *Klebsiella pneumoniae* (ATCC 132), and *Serratia marcescens* (ATCC 13880) were purchased from Carolina Biological Supply Company (Burlington, NC, USA) and stored as freezer stock cultures (nutrient broth:glycerol, 87.5∶12.5) at −80 °C.

Sinapinic acid was purchased from Sigma-Aldrich (St. Louis, MO, USA). Trifluoroacetic acid (TFA) and acetonitrile were purchased from ACROS (Fair Lawn, NJ, USA). MALDI calibrants were purchased from Sigma-Aldrich (St. Louis, MO, USA). Nutrient agar and nutrient broth were purchased from Carolina Biological Supply Company (Burlington, NC, USA).

### Parameter selection

Automated data acquisition is typically achieved using a software algorithm which requires the user to specify various parameters. These parameters control the laser power, peak evaluation, mass spectra accumulation, and laser movement on sample. Effects of these parameters on spectrum quality and reproducibility have not been thoroughly studied. Moreover, the values of some of these parameters have not been specified in the literature. For each of these reasons, we evaluated five parameters which are frequently reported in the literature [37]–[41]. The five factors which are adjusted through the Bruker FlexControl software (version 3.0; Bruker Daltonics) during automated data acquisition included: A) peak selection mass range; B) base peak S:N; C) base peak intensity; D) base peak minimum resolution; and E) number of shots summed (Table 1). Peak selection mass range defines the mass range for peak evaluation during automated data acquisition. The base peak is the highest peak observed in the peak selection mass range during automated data acquisition. The value of S:N, intensity and minimum resolution for the base peak must exceed the user-defined levels of these parameters; otherwise, the entire spectrum will be excluded during automated data acquisition.

**Table 1:** Factors and levels used in the fractional factorial experimental design.

		Level a		
Name	Factor	−1	0	+1
Peak selection mass range (kDa)^b^	A	1	4.5	8
S:N	B	1	2	3
Base peak intensity	C	100	200	300
Minimum resolution	D	100	250	400
Number of shots summed	E	100	300	500

a −1 represents the low level; 0 represents the center point; +1 represents the high level.

b Peak selection mass range represents a continuous mass range. The mid-point of the mass range was 10 kDa. The interval of the mass range was represented by an absolute value. Specifically, the peak selection value for level −1, 0 and +1 were 9 to 11 kDa, 5.5 to 14.5 kDa and 2 to 18 kDa, respectively.

### Fractional factorial experimental design

Factorial experimental designs are those which involve two or more factors of interest and where all possible combinations of the factor levels are tested. In this study, spectrum reproducibility was the response. Five independent factors (automated data acquisition parameters) were varied (Table 1). All five factors were numeric type (i.e., values represented by numbers) in the designed experiments.

Factorial-based designs can be separated into two main groups which are full factorial design and fractional factorial design [42]. Different types of factorial design yield different numbers of experiments. For example, if three factors are investigated and each factor has four levels which are to be tested, a full factorial design would require 64 (4^3^) experimental runs. As the number of factors and/or the number of levels of each factor increases, the experimental design becomes prohibitively large [36]. A full factorial design is generally used when the number of factors is small, for example, less than four [43]. If *k* factors could be set at two levels each, then a 2^k^ factorial design can be implemented. For three factors, each at two levels, a 2^3^ factorial design would require only 8 experimental runs. These designs are often used as screening designs to aid in identifying important factors and interactions [36]. Although using two levels of each factor is efficient, the experimental design can still become quite large as *k* increases. Therefore, a fractional factorial design denoted as 2*^k^* ^-*p*^ (k: the number of factors; p: the fraction index) was used in this study based on assumptions that higher-order interactions are negligible. Higher-order interactions are those which involve three or more factors. In this study, we focused on the interactions involving two factors. Because of the sparsity of effects principle [36], we assumed that the higher-order interactions were negligible and did not need to be estimated. As a result, a 2^5-1^ design was used. This yielded 16 experimental runs. It is important to note the 16 runs are not chosen at random or haphazardly. The 16-run orthogonal design is selected in order to eliminate confounding between main effects and minimize confounding between two-factor interactions [36].

The two levels for each factor were selected as they represent commonly used values [2], [18], [20], [44] and prior work in our lab [11]. Specifically, in the literature, many mass ranges have been used for peak selection, for example, 2 to 20 kDa [11], [18], [40], [41], [45], 2 to 6 kDa [37], 3 to 20 kDa [38], and 7 to 10 kDa [2]. Base peak S:N is usually set as 2 or 3 in literature [2], [11], [38]. Base peak intensity can vary from 100 to 600 [2], [11]. Few studies have specified base peak minimum resolution. In our previous work, this value was set at 400 [3], [11], [18]. The number of shots summed in different studies often varies from 100 to 1000 shots [2], [13], [38]–[41], [46]. Based on these reported values, two levels of each factor were selected and are shown in Table 1. The low level is designated as -1 and the high level is designated at +1 for coding purposes (Table 1).

In addition to the high and low levels of each factor, we also included center points (designated as 0) (Table 1) to assess whether the response changed linearly as the factor moved from its low to high level or if curvature in the response was present. Center points are those experiments where all five factors are set at their center value (Table 1). In this study, three center points were added, resulting in a total of 19 experiments (Table 2). The design of experiment software used in this study was Minitab Statistical Software (version 16) (Minitab Inc, PA, USA).

**Table 2:** Experimental design matrix of the fractional factorial designs and the resulting reproducibility of mass spectra.

	Factor					
Experiment	Peak selection range (kDa)	S:N	Threshold intensity	Minimum resolution	Number of shots summed	Interreplicate imilarity (%)^a^
1	1	1	300	400	500	95.5±3.4
2	8	3	100	400	100	86.6±7.9
3	8	1	300	100	500	97.0±1.4
4	8	3	300	100	100	92.1±3.4
5	1	3	300	400	100	87.6±6.0
6	1	3	300	100	500	97.7±0.9
7	1	1	100	100	500	98.0±0.9
8	1	3	100	100	100	92.4±2.3
9	8	1	100	400	500	96.3±1.5
10	8	3	300	400	500	97.9±1.1
11	4.5	2	200	250	300	95.2±3.4
12	8	3	100	100	500	96.5±2.3
13	8	1	300	400	100	91.5±4.4
14	1	3	100	400	500	96.3±2.3
15	1	1	100	400	100	87.1±7.7
16	4.5	2	200	250	300	94.3±2.7
17	4.5	2	200	250	300	95.4±1.8
18	1	1	300	100	100	92.9±5.3
19	8	1	100	100	100	92.8±3.0

a Values reported are the average correlation coefficients of 10 replicates ± the standard deviations of the correlation coefficient.

### Sample preparation

A nutrient agar plate was streaked from freezer stock and incubated at 37 °C for 24 hours. A single colony was inoculated into 5 ml sterile nutrient broth, and the broth was incubated at 37°C for 24 hours on an orbital shaker at 200 rpm. Samples were prepared as previously described [11]. Briefly, 1 ml of culture (O.D. _600_ = 0.8) was centrifuged at 14,000 × g for 5 minutes. After removal of the supernatant, the cell pellet was resuspended in 1 ml of sterile double-distilled water (ddH_2_O) (Millipore Corp.; Bedford, MA, USA) and centrifuged again at 14,000 × g for 5 min. The supernatant was decanted and the resulting cell pellet was resuspended in 100 μl of sterile ddH_2_O. Sinapinic acid matrix solution was prepared as previously described [11]. Equal volumes of cell suspension and matrix solution were mixed. Aliquots (2 μl) of this mixture were spotted onto a MSP 96 ground steel target plate (Bruker Daltonics; Billerica, MA, USA) and allowed to air dry.

### MALDI-TOF MS analysis

MALDI-TOF MS analyses were performed using a Bruker Microflex LRF MALDI-TOF mass spectrometer (Bruker Daltonics; Billerica, MA, USA) equipped with a nitrogen laser (λ = 337 nm) under the control of FlexControl software (version 3.0; Bruker Daltonics). Mass spectra were automatically collected in positive linear mode with varying combinations of automated data acquisition parameter values (Table 2). Ion source 1 voltage was set to 20 kV, ion source 2 voltage was set to 18.15 kV, and the lens voltage was set to 9.05 kV. Other parameters were set as described previously [11]. Mass calibration was performed using standard calibrants: ACTH (1–17) 2094.427 Da, ACTH (18–39) 2466.681 Da, Insulin oxidized B 3494.6513 Da, Insulin 5734.518 Da, Cytochrome C 12360.974 Da and Myoglobin 16952.306 Da (Sigma-Aldrich, St. Louis, MO, USA).

Raw spectra were post-processed and peaks were picked using FlexAnalysis software (version 3.0; Bruker Daltonics). Masses from 2 to 20 kDa were used for spectrum evaluation and post-processing. Minimum peak resolution was set at 400 Da. The minimum S:N threshold was set at 2, while the minimum peak intensity threshold was set at 100. Baseline subtraction was performed using the TopHat algorithm [47].

### Quantification of spectrum quality and reproducibility

Measures of spectrum quality included base peak intensity, base peak resolution, base peak S:N, number of peaks, and mass range. To quantify reproducibility, peak lists generated by FlexAnalysis were imported into BioNumerics software (version 6.1; Applied Maths; Sint-Martens-Latem, Belgium) using a custom script created by the manufacturers of the software for this application. Similarity coefficients of replicate spectra were calculated using the Pearson product-moment correlation coefficient [48].

### Statistical analysis

Each of the 19 runs from the designed experiments (Table 2) consisted of 5 technical replicates of *P. aeruginosa*. All 19 experiments were carried out on the same day in a randomized order and distribution on the MALDI target, resulting in 95 mass spectra. These spectra constituted one dataset. In total, two datasets were obtained on two consecutive days. Both datasets were subjected to analysis of reproducibility, spectrum quality, main effects, and interactions of factors. Specifically, reproducibility and spectrum quality of each designed experiment were reported using the averaged values of 10 replicates of *P. aeruginosa* from the two datasets. Main effects and interactions of factors on reproducibility were analyzed based on analysis of variance (ANOVA) and t-tests using a 5% level of significance [36] (Minitab Inc, PA, USA).

### Optimization

Most optimization efforts using univariate approaches have not evaluated optimized experimental conditions beyond the species or strains that undergo optimization. We hypothesized that the optimized settings may improve spectrum quality and reproducibility of spectra from bacteria other than *P. aeruginosa*. Therefore, two other gram negative bacteria, *Klebsiella pneumoniae* and *Serratia marcescens*, both of which showed low reproducibility when using non-optimized settings [11], were also analyzed via MALDI using optimized settings. For either optimized or non-optimized settings, 20 spectra were acquired representing 20 technical replicates for each bacterium. The reproducibility and quality of spectra from each bacterium before and after optimization were reported using the averaged values of the corresponding 20 mass spectra. Differences in spectrum quality and reproducibility before and after optimization were identified using t-tests with a 5% level of significance (Minitab Inc, PA, USA).

## Results and Discussion

### Design matrix and reproducibility

The highest reproducibility achieved for *P. aeruginosa* using optimized automated data acquisition was 98.0% (Table 2), which is higher than the previously reported value (88.3%) (p-value
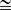
0.001) for non-optimized automated data acquisition, and was comparable to the reproducibility (96.1%) obtained manually [11]. The corresponding experimental settings for this high reproducibility were: peak selection mass range  = 9 to 11 kDa, S:N = 1, base peak intensity  =  100, base peak minimum resolution  =  100, and number of shots summed  =  500. In contrast, low reproducibility was also observed in these 19 experiments, ranging from 86% to 88%, which was comparable to the previously reported value (88.3%) for non-optimized automated data acquisition [11]. These results show clearly that the values of parameters used in the automated data acquisition procedure influence reproducibility.

### Spectrum reproducibility and quality

**Figure 1:**
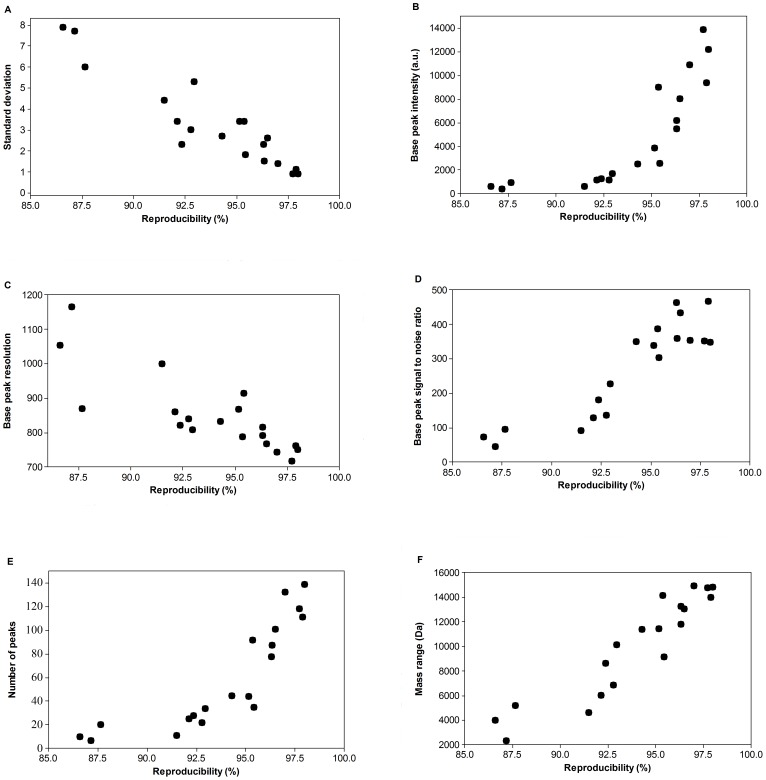
Characterization of spectra exhibiting varying levels reproducibility. Metrics of quality include standard deviation (A), base peak intensity (B), base peak resolution (C), base peak S:N (D), number of peaks (E), and mass range (F).

To further investigate spectrum quality and reproducibility obtained using different automated data acquisition settings, we assessed metrics of spectrum quality as a function of reproducibility for all 19 experiments. Specifically, we examined spectra exhibiting varying levels of reproducibility with regard to the standard deviation of the reproducibility and their spectrum quality, including base peak intensity, base peak resolution, base peak S:N, number of peaks, and mass range (Fig. 1).

As expected, our analysis revealed that spectra with higher reproducibility tended to have lower standard deviations (Fig. 1A). With regard to spectrum quality, spectra with higher reproducibility tended to have higher base peak intensities (Fig. 1B), higher base peak S:N (Fig. 1D), greater numbers of peaks (Fig. 1E), and broader mass ranges (Fig. 1F). These results indicate that highly reproducible spectra are associated with high spectrum quality.

Interestingly, we observed a counterintuitive relationship between base peak resolution and reproducibility. Highly reproducible spectra tended to have lower resolution base peaks than spectra with lower reproducibility (Fig. 1C). While base peak resolution is an important parameter to assess spectrum quality (high resolution is typically desired), our results suggest that spectra with high reproducibility more commonly had lower base peak resolutions. To investigate the possibility that our result was based on anomalous spectra, we manually and rigorously examined each spectrum to ensure each spectrum contained at least 5 peaks which had intensities higher than 100 arbitrary units. These results suggest that efforts to increase base peak resolution when optimizing MALDI-TOF settings may not necessarily increase spectrum reproducibility. Our results further suggest that a conventional standard for assessing spectrum quality, base peak resolution, may have more limited applicability to microbial characterization via MALDI than to more traditional applications of mass spectrometry (e.g., protein identification). Accordingly, future attempts to optimize automated data acquisition should not place undue emphasis on base peak resolution.

### Effects of automated data acquisition parameters on reproducibility

**Figure 2:**
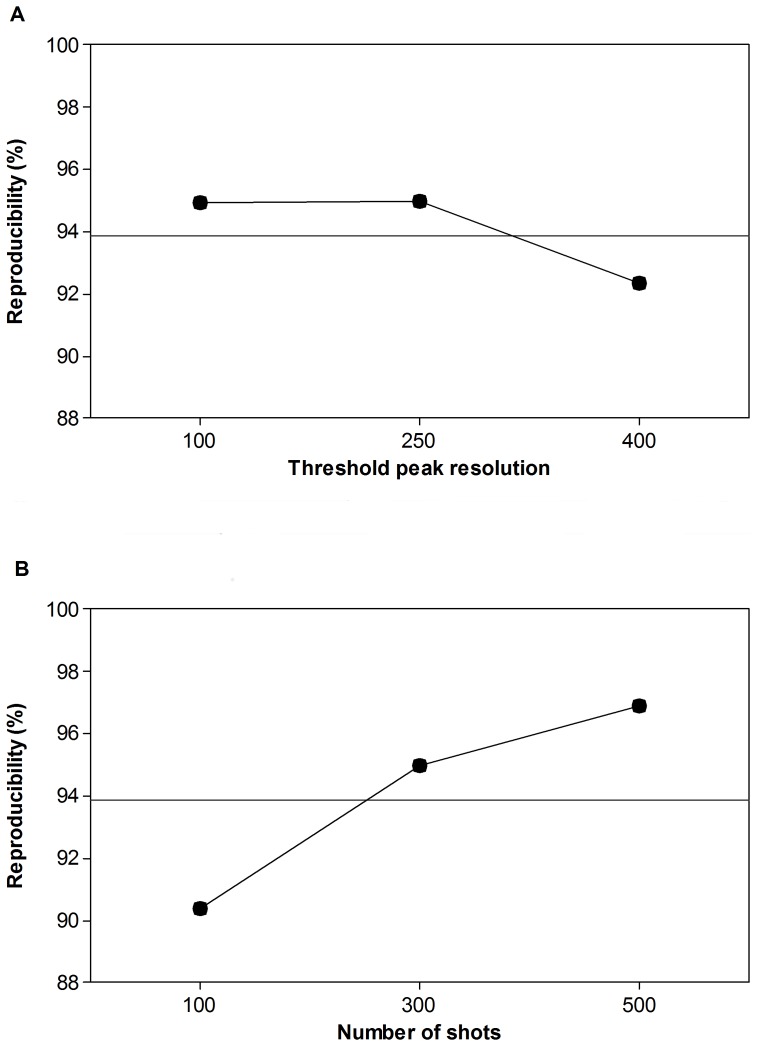
Main effects plots. Effects of threshold minimum resolution (A) and number of shots summed (B) on spectrum reproducibility during characterization of *P. aeruginosa* using automated data acquisition with MALDI-TOF MS.

Statistical analysis was used to identify main effects and two-factor interaction effects of automated data acquisition parameters on reproducibility. The estimated effect for any factor or interaction is the difference between the average response at the high level of that factor or interaction and its low level. For example, the estimated effect of factor A would be (if 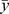 represents the response of interest): 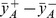
_._ The plus and minus superscripts represent values of the responses at the high and low levels, respectively. If this difference is large (in absolute value) then factor A would be considered statistically significant. The analysis of variance results are displayed in Table 3. Factors and interactions that had a p-value less than 0.05 were considered significant. Based on the p-values, threshold peak resolution (D) (p-value
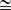
0.013) and number of shots summed (E) (p-value
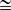
0.002) were found to be significant. The main effects are shown in Figure 2. The mean value of reproducibility obtained with the high level of threshold resolution decreased in comparison with that obtained with the low level of threshold resolution (Fig. 2A). It is also illustrated in Figure 2 that the effect of number of shots on reproducibility shows a positive trend, in which spectrum reproducibility increased with the number of shots summed (Fig. 2B).

**Table 3:** Analysis of variance for reproducibility.

Source^a^	DF	Seq SS	Adj SS	Adj MS	F	P
Main effects	5	200.105	200.105	40.021	112.51	0.009
A	1	0.643	0.643	0.643	1.81	0.311
B	1	0.951	0.951	0.951	2.67	0.244
C	1	2.380	2.380	2.380	6.69	0.123
D	1	26.631	26.631	26.631	74.87	0.013
E	1	169.501	169.501	169.501	476.52	0.002
Two-way interactions	10	28.278	28.278	2.828	7.95	0.117
A * B	1	1.596	1.596	1.596	4.49	0.168
A * C	1	2.593	2.593	2.593	7.29	0.114
A * D	1	4.488	4.488	4.488	12.62	0.071
A * E	1	0.398	0.398	0.398	1.12	0.401
B * C	1	0.072	0.072	0.072	0.20	0.696
B * D	1	0.001	0.001	0.001	0.00	0.965
B * E	1	3.430	3.430	3.430	9.64	0.090
C * D	1	2.162	2.162	2.162	6.08	0.133
C * E	1	1.253	1.253	1.253	3.52	0.201
D * E	1	12.285	12.285	12.285	34.54	0.028
Curvature	1	4.399	4.399	4.399	12.37	0.072
Residual error	2	0.711	0.711	0.356		
Pure error	2	0.711	0.711	0.356		
Total	18	233.494				

a A, B, C, D and E represent peak selection mass range, S:N, base peak intensity, minimum resolution, and number of shots summed, respectively.

**Figure 3:**
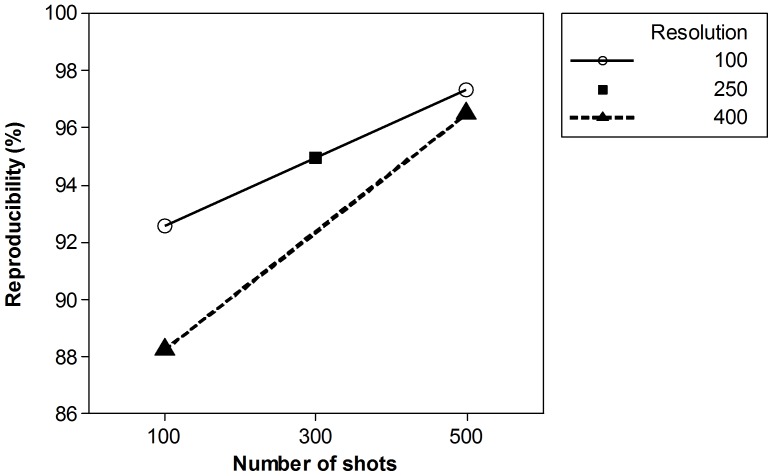
Interaction of threshold minimum resolution and number of shots summed. Effects on spectrum reproducibility during characterization of *P. aeruginosa* using automated data acquisition with MALDI-TOF MS.

An interaction between minimum resolution and number of shots summed (D*E) (p-value 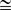 0.028) was observed (Fig. 3) indicating that the number of shots summed is more important in the case of higher threshold resolution (e.g., 400). In contrast, when using a lower threshold resolution, for example 100, fewer shots appeared to yield reproducibility comparable to that obtained using more shots and a higher threshold resolution (Fig. 3). This finding is intriguing, because it suggests that fewer shots may be used to obtain spectra of reproducibility comparable to that obtained with many more shots. Reducing shot number has the potential to reduce the time required for analysis. This might be particularly valuable information in a clinical microbiology lab setting in which the number of samples processed per day is very high.

A prediction equation (Eq. 1) was fitted for *P. aeruginosa* to predict reproducibility for each experimental run, where ŷ is predicted reproducibility (%), D is threshold minimum resolution and E is number of shots summed.
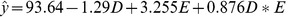
(Eq. 1)


Based on the interaction plot (Fig. 3), setting the number of shots at 500 and resolution at 100 yielded an overall higher average reproducibility than any other combination of the two factors. A response optimization algorithm was also used to find best combinations of threshold minimum resolution and number of shots summed for high reproducibility. This showed the same settings as the interaction plot suggested (data not shown). As a result, we input the threshold minimum resolution at its low level and number of shots summed at its high level, which were −1 and +1, respectively, into the fitted equation (Eq.2). As shown in Eq. 2, the predicted reproducibility for *P. aeruginosa* was 97.3%.
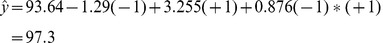
(Eq. 2)


### Effects of optimization on automated data acquisition

Finally, we compared spectrum quality and reproducibility using non-optimized and optimized automated data acquisition settings. The non-optimized settings were previously described [11], in which peak selection ranged from 2 to 20 kDa; S:N was 2; base peak intensity was 100; minimum resolution was 400 and number of shots summed was 300. The optimized settings were those used in Eq. 2 as described above.

**Figure 4:**
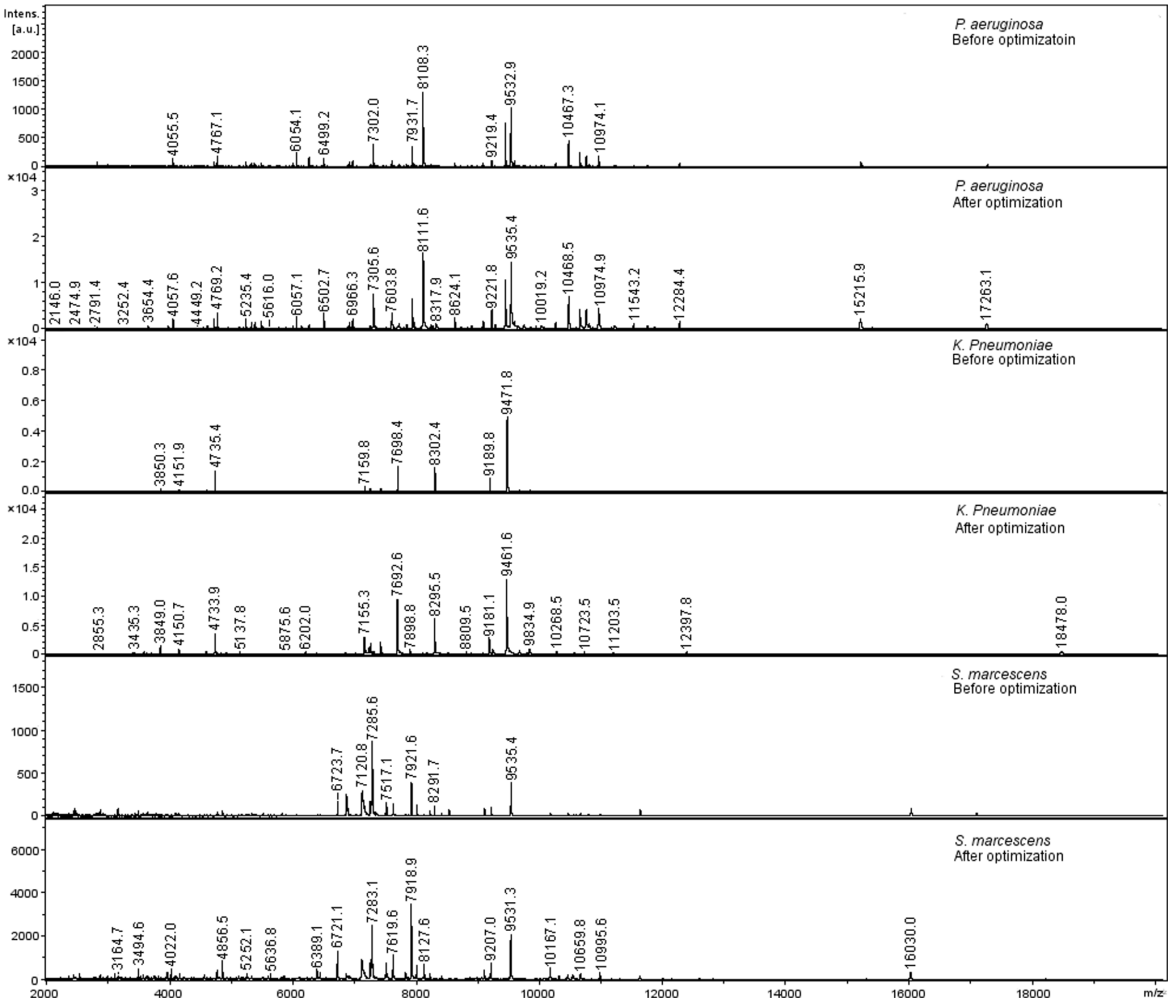
Representative mass spectra acquired via non-optimized and optimized automation settings. Mass spectra of *P. aeruginosa*, *K. pneumoniae* and *S. marcescens* were acquired via non-optimized and optimized settings using automated data acquisition with MALDI-TOF MS.

Representative mass spectra obtained before and after optimization are shown in Figure 4, and corresponding spectrum quality and reproducibility metrics are summarized in Table 4. Generally, base peak intensity, number of peaks, and mass range increased when optimized data acquisition settings were used for all three bacteria (Table 4; Fig. 4). No difference was observed for S:N between non-optimized and optimized settings. With regard to base peak resolution, spectra obtained using optimized settings had a lower base peak resolution than those obtained using non-optimized settings for all three bacteria.

**Table 4:** Effect of optimization on spectrum quality and reproducibility.

		Spectrum quality^a^					
Bacteria	Condition	Base peak intensity	Base peak resolution	Base peak S:N	Number of peaks	Mass range (Da)	Reproducibility (%)^b^
*P. aeruginosa*	Before optimization	2143.5± 1623.5*	869.2±134.1**	224.6±127.8*	40.6±28.9*	9102.6±4040.6 *	90.4±5.5*
	After optimization	13751.8±4907.3**	718.5±71.6*	259.0±96.7*	93.6±35.2**	14649.4±650.4**	97.2±1.2**
	*p* value Pr >| t |	0.000	0.000	0.344	0.000	0.000	0.000
*K. pneumoniae*	Before optimization	3859.7±1641.1*	920.9±83.3**	585.1±206.0*	27.6±24.9*	6188.0±1498.7*	93.6±5.1*
	After optimization	21431.4±3387.6**	776.1±59.0*	660.6±153.6*	152.1±28.7**	15734.8±400.1**	97.5±1.8**
	*p* value Pr >| t |	0.000	0.000	0.197	0.000	0.000	0.000
*S.marcescens*	Before optimization	1543.9±919.2*	957.7±232.7**	121.1±37.1*	38.8±22.1*	7126.2±4091.3*	84.6±9.9*
	After optimization	8333.4±2137.1**	750.3±51.6*	100.7±27.2*	30.3±18.7*	12087.2±1410.4**	93.9±2.9**
	*p* value Pr >| t |	0.000	0.000	0.056	0.199	0.000	0.000

a Values reported are the means of 20 replicates ± the standard deviations of the mean. Data were analyzed by t test (α = 0.05).

b Values reported are the average correlation coefficients of 20 replicates ± the standard deviations of the correlation coefficient.

Before and after optimization values for each bacterium followed by different numbers of asterisks are significantly different.

**Figure 5:**
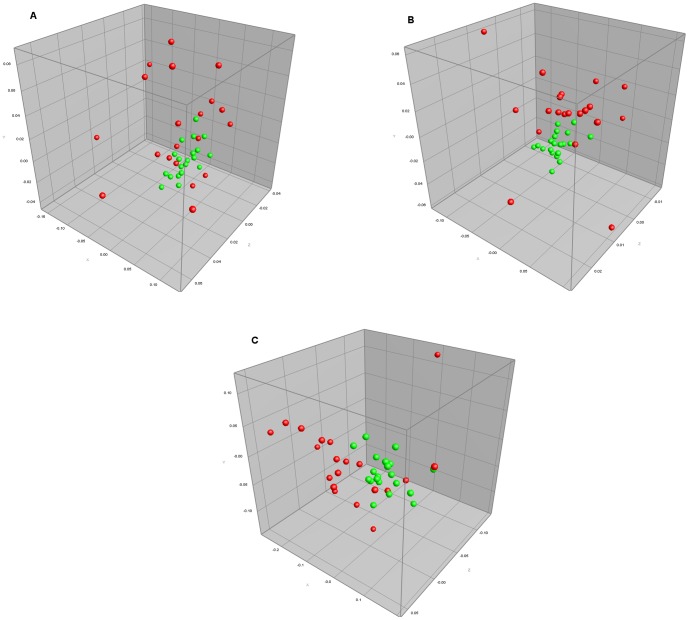
Multidimensional scaling (MDS) representation of spectrum reproducibility. MDS representing reproducibility associated with spectra of *P. aeruginosa* (A), *K. pneumoniae* (B) and *S. marcescens* (C) acquired via non-optimized (red) and optimized (green) settings.

Optimization increased spectrum reproducibility (Table 4). For example, reproducibility for *P. aeruginosa, K. pneumoniae* and *S. marcescens* before optimization was 90.4±5.5%, 93.6±5.1% and 84.6±9.9%, respectively, while after optimization, reproducibility was 97.2±1.2%, 97.5±1.8% and 93.9±2.9%, respectively (Table 4). Multidimensional scaling (MDS) was used to visualize effects of optimization on reproducibility (Fig. 5). For all three bacteria, replicate spectra (20 replicates for each bacterium) acquired using optimized settings grouped more closely than replicate spectra (20 replicates for each bacterium) acquired without optimization.

The reproducibility (97.2%) of *P. aeruginosa* using optimized settings was strikingly similar to that predicted using the fitted equation (97.3%). Values of peak selection mass range, S:N and threshold peak intensity can have multiple selections. Other selections of these three parameters with constant values of threshold minimum resolution (100) and number of shots summed (500) also yielded spectra with reproducibility comparable to predicted values (data not shown).

We further compared the reproducibility obtained using optimized automated data acquisition settings with reproducibility previously reported which was obtained from spectra acquired manually [11]. They were comparable for all three bacteria. Specifically, reproducibility reported previously for manual data acquisition was 96% to 97% for *P. aeruginosa*, 95% to 96% for *K. pneumoniae* and 93% to 96% for *S. marcescens* [11]. For automated data acquisition using optimized settings, the reproducibility was approximately 97% for *P. aeruginosa*, 98% for *K. pneumoniae* and 94% for *S. marcescens* (Table 4).

The optimized settings were effective in increasing spectrum reproducibility for bacteria beyond the one that served as the model for optimization, suggesting that these settings, to some extent, are effective in improving the reproducibility of spectra for a range of bacteria. However, our model and equation are based on data acquired using *P. aeruginosa*, a Gram-negative bacterium. With regard to other bacteria, particularly Gram-positive bacteria, the relevance of settings obtained here may have limited utility, and coefficients of models may need to be adjusted. Accordingly, it may be necessary to run designed experiments for specific strains to obtain unique optimum settings. Conversely, such optimization may not always be necessary. For example, Mellmann et al. 2009 [49] reported high reproducibility using parameters for automated data acquisition that had not been rigorously optimized for the bacteria characterized in that work.

## Conclusions

A fractional factorial design was applied to optimize five data acquisition parameters (peak selection mass range, S:N, threshold peak intensity, threshold minimum resolution and number of shots summed) and one response (reproducibility of replicate spectra). Both threshold minimum resolution and number of shots summed affected reproducibility, and an interaction was observed between these two data acquisition parameters. In the case of low threshold minimum resolution, high reproducibility could be achieved with fewer shots. After optimization, reproducibility of replicate spectra approached/exceeded those obtained manually for *P. aeruginosa*, *K. pneumoniae* and *S. marcescens*, suggesting that the main effects and interaction found in this study may be applicable to a broad range of bacteria. To our knowledge, this is the first report of use of designed-experiments to optimize automated data acquisition during MALDI-TOF fingerprint-based experiments.
